# Circular RNAs Repertoire and Expression Profile during *Brassica rapa* Pollen Development

**DOI:** 10.3390/ijms221910297

**Published:** 2021-09-24

**Authors:** Saeid Babaei, Mohan B. Singh, Prem L. Bhalla

**Affiliations:** Plant Molecular Biology and Biotechnology Laboratory, Faculty of Veterinary and Agricultural Sciences, The University of Melbourne, Parkville, Melbourne, VIC 3010, Australia; sbabaeighara@student.unimelb.edu.au (S.B.); mohan@unimelb.edu.au (M.B.S.)

**Keywords:** *Brassica rapa*, circRNAs, pollen development, RNA sequencing

## Abstract

Circular RNAs (circRNAs) are covalently closed RNA molecules generated by the back-splicing of exons from linear precursor mRNAs. Though various linear RNAs have been shown to play important regulatory roles in many biological and developmental processes, little is known about the role of their circular counterparts. In this study, we performed high-throughput RNA sequencing to delineate the expression profile and potential function of circRNAs during the five stages of pollen development in *Brassica rapa*. A total of 1180 circRNAs were detected in pollen development, of which 367 showed stage-specific expression patterns. Functional enrichment and metabolic pathway analysis showed that the parent genes of circRNAs were mainly involved in pollen-related molecular and biological processes such as mitotic and meiotic cell division, DNA processes, protein synthesis, protein modification, and polysaccharide biosynthesis. Moreover, by predicting the circRNA–miRNA network from our differentially expressed circRNAs, we found 88 circRNAs with potential miRNA binding sites, suggesting their role in post-transcriptional regulation of the genes. Finally, we confirmed the back-splicing sites of nine selected circRNAs using divergent primers and Sanger sequencing. Our study presents the systematic analysis of circular RNAs during pollen development and forms the basis of future studies for unlocking complex gene regulatory networks underpinning reproduction in flowering plants.

## 1. Introduction

**Figure 1:**
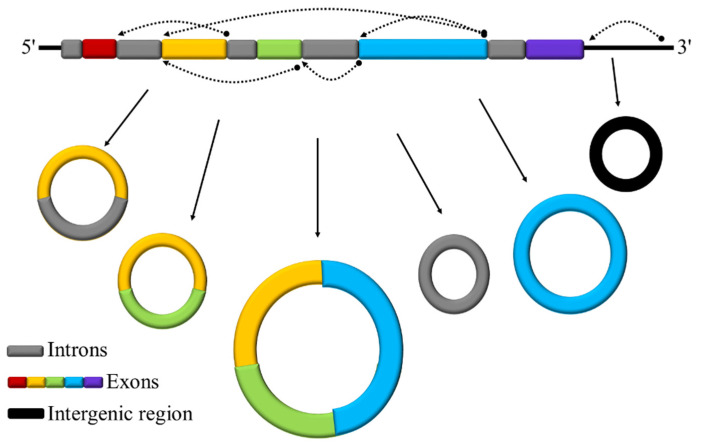
A schematic representation of back-splicing producing basic types of circRNAs from different genomic regions. Dashed curved arrows represent back-splicing junctions.

Transcriptome-wide sequencing studies have shown that the genomic information is extensively transcribed into protein-coding RNAs and RNAs with no protein coding potential but play widely important regulatory roles. Although alternative splicing and post-transcriptional events can generate numerous mRNA isoforms to increase the protein-coding and regulatory capacity of the eukaryotic genomes, these RNAs comprise only a small percentage (usually about 1–2%) of all RNAs in the cell [1]. The majority of expressed RNAs are non-coding RNAs that, based on their size, can be divided into categories, such as small non-coding and long non-coding RNAs (lncRNAs) [2,3]. These non-coding RNAs have been known to be key regulatory factors controlling gene expression at the post-transcriptional, transcriptional, and epigenetic levels [4]. CircRNAs are another layer of transcriptome complexity that came into focus about a decade ago and are considered non-coding RNAs; however, the protein-coding ability for a subset of them has been reported in recent studies [5,6]. CircRNAs are covalently closed single-stranded RNA molecules (with no 5′ caps or 3′ poly-A tails) following transcription from a wide range of genomic positions, including exons, introns, and intergenic regions, that undergo a non-canonical splicing event termed as back-splicing [7] (Figure 1). In humans and animals, it was revealed that base pairing from reverse complementary sequences in the flanking introns of circularized exons, as well as some RNA-binding proteins such as Quaking (QKI), promote circRNA production [8]. However, the molecular mechanism behind back-splicing in plants is still poorly understood [9,10]. Despite being known for decades, circRNAs have been considered as aberrant byproducts of mis-splicing events [10]. With the advances in RNA-seq technology and bioinformatics algorithms, thousands of circRNAs have been identified in the eukaryotic tree of life and revealed to have cell- and tissue-specific expression patterns [9,11]. Earlier studies have demonstrated that lncRNAs play important roles in reproductive-related processes such as floral transition and flower development [12]. Cold-Assisted Intronic Noncoding RNA (*COLDAIR*) and Cold-Induced Long Antisense Intragenic RNA (*COOLAIR*) are lncRNAs that control flowering in *Arabidopsis* by repressing Flowering Locus C (*FLC*) through epigenetic mechanisms [13,14]. In rice, it has been revealed that more than 700 long intergenic non-coding RNAs are essential factors for the biogenesis of phased small interfering RNAs that associate with germline-specific Argonaute protein MEL1, which itself is involved in the development of pre-meiotic germ cells, suggesting that rice lncRNAs are important elements in reproduction [15].

Cell-specific expression of thousands of protein-coding transcripts underpins pollen developmental processes [16,17,18,19]. *Cis*-regulatory elements, tissue-specific promoters, histone modifications, and DNA methylation have also been reported to be involved in the regulatory network of pollen development [20,21,22,23,24]. LncRNAs have also been implicated in controlling pollen reproductive developmental processes [12]. For example, Long-Day-Specific Male-Fertility-Associated RNA (*LDMAR*) and *Zm401* are lncRNAs required for pollen development in rice and maize, respectively [25,26]. A recent study in *Brassica rapa* found that more than 12,000 lncRNAs were expressed during pollen development and fertilization [27]. In addition, recent studies have implicated circRNAs in the regulation of pollen development. For instance, in rice, 186 differentially expressed circRNAs have been identified in different pollen developmental stages during the fertility transition of the photo-thermosensitive genic male sterile line [28]. In soybean, 2867 circRNAs were identified in flower buds of the cytoplasmic male sterile line (NJCMS1A) and its maintainer (NJCMS1B), of which 1009 circRNAs were differentially expressed between two lines, pointing towards the role of circRNAs in flower and pollen development [29]. Similar work in *B. campestris* revealed 31 differentially expressed circRNAs between cytoplasm male sterile and fertile lines involved in anther development [30]. To further improve our understanding of the role of circRNAs in pollen development, we performed a time series of RNA-seq experiments during pollen development in *B. rapa* (AA, 2n = 20). Using three different bioinformatics prediction tools, we identified 1180 circRNAs expressed in five pollen developmental stages. We performed differential gene expression analysis, functional annotation, and pathway enrichment analysis to explore the potential function of circRNAs during pollen development. We also predicted miRNA–circRNA interactions to investigate the potential role of circRNAs as competing endogenous RNAs (ceRNAs) in post-transcriptional gene regulation.

## 2. Materials and Methods

### 2.1. Plant Materials, Growth Conditions, and Sample Collection

In this study, *Brassica rapa*, accession no. ATC 92270 Y.S (AND)-168, were grown at 21/18 °C day/night under 16/8 h’ light/dark (200 μmol m^−2^ s^−1^ light intensity) with 60% humidity. Based on the size of floral buds, five pollen developmental stages were identified: pollen mother cells: ≤1 mm buds, tetrad: 1.2–2 mm buds, uninucleate pollen: 2–2.6 mm buds, binucleate pollen: 3–4 mm buds, and mature pollen: 5–6 mm buds (Figure S1) [27]. The samples were pooled from 15 different plants for each stage of pollen development. Collected buds were immediately transferred to a Petri dish containing modified half strength B5 medium (13% sucrose) on ice. Pollen from the buds in their binucleate and mature pollen stages was released by dissecting out the anthers and squashing them in the modified B5 medium. Because the buds in the first three groups (pollen mother cells, tetrad, and uninucleate pollen) were too small, the whole buds were squashed in the modified B5 medium to release the pollen. The resulting suspensions were then filtered using a 40 µm mesh (pluriStrainer Mini 40µm, pluriSelect, Leipzig, Germany) into 2 mL tubes. Tubes were then centrifuged at 4 °C for 3 min at 150 g, and the supernatant was discarded. The pellet was washed with modified half strength B5 medium, and finally, pure pollen grains were collected from the medium using centrifugation at 4 °C for 3 min at 150 g. After discarding the medium, the pellet was immediately frozen in liquid nitrogen and stored at −80°C for later use [31].

### 2.2. RNA Extraction and Sequencing

The total RNA was isolated from collected pollen samples using the mirVana™ miRNA Isolation Kit (Thermo-Fisher; Part Numbers AM1560, AM1561, Carlsbad, CA, USA) according to the manufacturer’s instructions. To remove contaminated DNA, all the isolated RNA samples were treated with TURBO™ DNase (Ambion, Carlsbad, CA, USA) and then submitted for sequencing. The RNA-seq libraries were then constructed using Illumina TruSeq stranded kit following the rRNA depletion step, and the single-end reads (100 bp) sequencing was performed at the Australian Genome Research Facility (AGRF), Melbourne. The sequencing data were deposited to NCBI’s Sequence Read Archive (SRA) under accession number PRJNA763698.

### 2.3. Identification and Differential Expression of Circular RNAs

After obtaining clean reads by removing low-quality and adaptor sequences, reads were mapped to the *B. rapa* reference genome (v3.0, http://brassicadb.org/brad/, accessed on 29 July 2020) [32] using BWA (v0.7.17, mem-T19) [33], Bowtie2 (v2.3.5.1) [34], and STAR (v2.7.5a) [35] due to the fact that circRNA identification tools work with different aligners. Then, circRNAs were identified using the top three identification tools [36], CIRI2 (v2.0.6) [37], find_circ (v1.2) [38], and CIRCexplorer2 (v2.3.8) [39] with their default parameters. After combining the results, the repeated circRNAs identified by two or more detection tools were removed. A list of unique circRNA candidates with at least two supported back-spliced reads was considered for subsequent analysis. Bedtools (v2.30.0) [40] was used to annotate genomic regions of identified circRNAs using the annotation file obtained from the *Brassicaceae* database (v3.0, http://brassicadb.org/brad/, accessed on 29 July 2020). The R-Bioconductor package Noiseq (v2.28.0) [41] with the TMM (TrimmedMean of M values) [42] normalization method was used for the quantification of expression level and detection of differentially expressed circRNAs between samples; circRNAs with the *q* value ≥ 0.8 were considered differentially expressed. Plots were generated using the R (v4.0.4) [43] with ggplot2 (v3.3.3) library [44]. The heatmap was generated using gplot (v3.1.1) [45].

### 2.4. Conservation Analysis of CircRNAs

For conservation analysis, the information of circRNAs identified in other plants (Table 1) was retrieved from PlantcircBase (release 6.0: http://ibi.zju.edu.cn/plantcircbase/, accessed on 29 July 2021), and then BLASTN (-word_size 11, -evalue 1e-5) [46] was used to compare 1180 circRNAs detected in this study against those from other plant species.

### 2.5. Functional Enrichment Analysis

Gene Ontology (GO) enrichment analysis was undertaken for functional categorization of the parental genes of differential expressed circRNAs by topGO package (v2.42.0) [47], and KEGG (Kyoto Encyclopedia of Genes and Genomes) pathway enrichment analysis was conducted by submitting the sequence of differentially expressed circRNAs in KOBAS database (v3.0, http://kobas.cbi.pku.edu.cn/kobas3, accessed on 21 August 2020) [48]. ClueGO (v2.5.8) [49] was used to summarize the most significant GO terms using “preselected Functions”.

### 2.6. Prediction of Potential circRNA–miRNA Interactions and Visualization

The potential interaction between known miRNAs and our identified differential expressed circRNAs was carried out using web tool psRNATarget (v2, http://plantgrn.noble.org/psRNATarget/home, accessed on 21 August 2020) [50] with default parameters, except we set the Expectation value to 3.0. We selected “*Brassica rapa*, 157 published miRNA” as input miRNAs and the sequence of our identified differentially expressed circRNAs as the target sequences. The circRNA–miRNA network was visualized using Cytoscape software (v3.8.2) [51]. We also predicted the mRNA targets of miRNAs using Targetfinder [52]. Then, GO enrichment analysis and KEGG pathway enrichment analysis were performed on predicted targets as described in Section 2.5.

### 2.7. Circular RNA Validation

To validate our identified circRNAs that were predicted by bioinformatic approaches, PCR with divergent primers followed by Sanger sequencing was used. Briefly, total RNA from all five pollen developmental stages was reverse transcribed into complementary DNA (cDNA) using SuperScript™ III Reverse Transcriptase (Invitrogen, Carlsbad, CA, USA) and random hexamers according to the manufacturer’s protocol. The sequences of 15 in silico predicted circRNAs that were predicted to be expressed in developing pollen were used to design 15 pairs of divergent primers using Primer3web (v4.1.0) [53]. The PCR procedure was as follows: initial step at 94 °C for 3 min; followed by 40 cycles at 94 °C for 30 s, appropriate annealing temperature (Table S1) for 45 s, and 72 °C for 30 s; and then 1 cycle at 72 °C for 7 min. The PCR products were then visualized by agarose gel (1.5%) electrophoresis, and the desired bands were recovered from the gel using Wizard^®^ SV Gel and PCR Clean-Up System (Promega, Madison, WI, USA). Finally, the PCR products were cloned into a pJET1.2/blunt vector using the CloneJET PCR Cloning Kit (Thermo Scientific, Vilnius, Lithuania) and subjected to Sanger sequencing to confirm the back-spliced junction sites.

## 3. Results

### 3.1. Identification and Characterization of circRNAs

**Figure 2:**
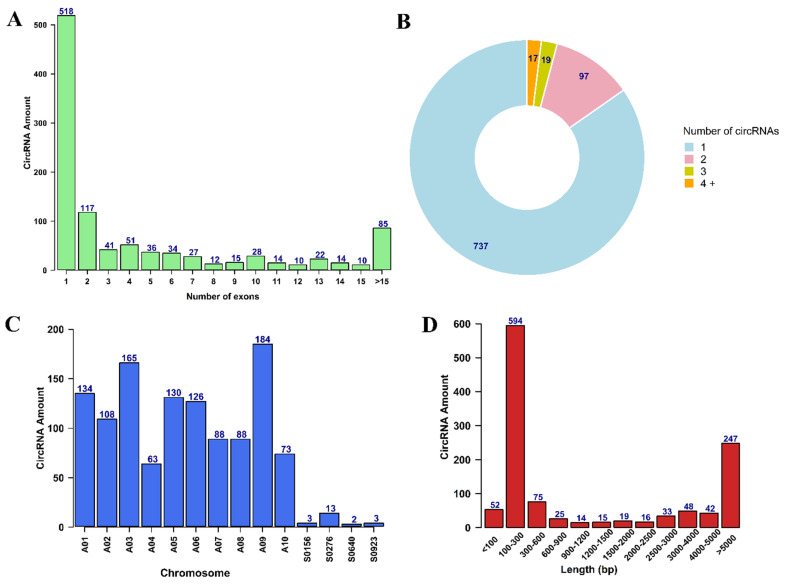
Genomic features of identified circRNAs during pollen development in *B. rapa*. (**A**) Distribution of the exon number per detected circRNA. (**B**) Distribution of the number of circRNA reads generated from the same parental gene. (**C**) The number of circRNAs detected in each chromosome. (**D**) Distribution of the length of circRNAs.

**Figure 3:**
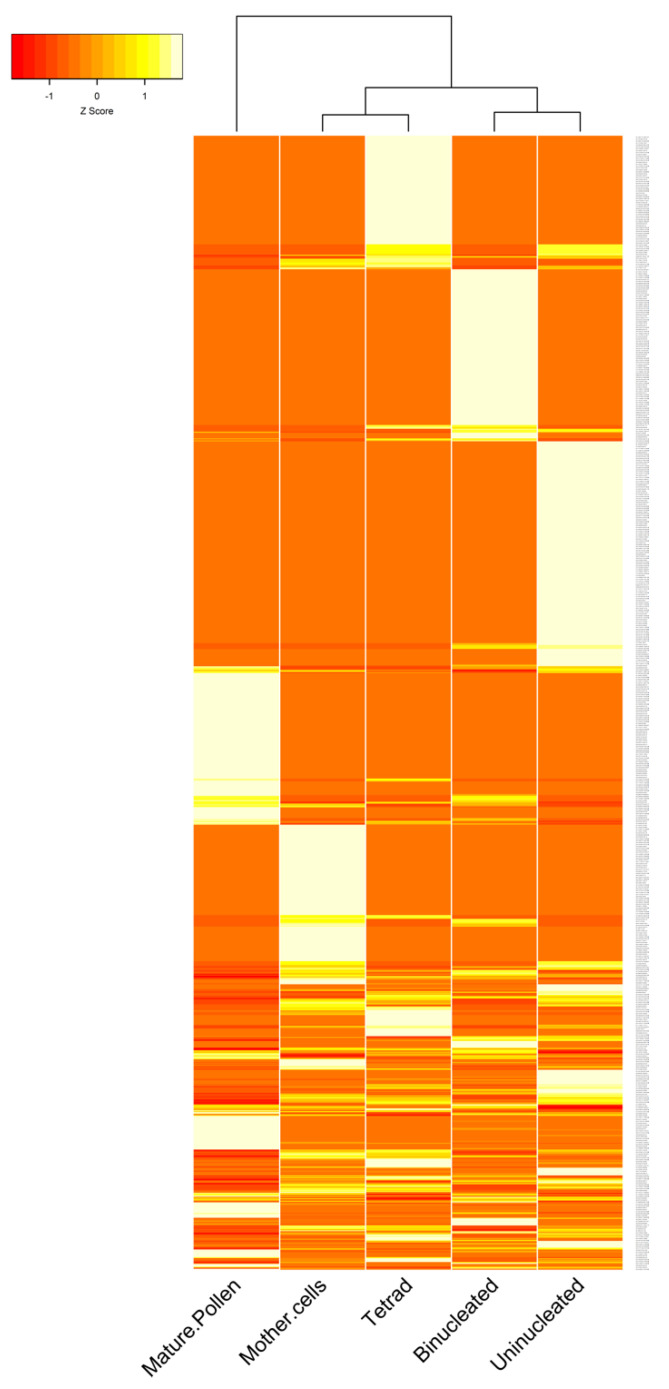
A heatmap showing the expression of 1180 identified circRNAs during pollen development in *B. rapa*.

To explore the involvement of circRNAs in *B. rapa* pollen development, rRNA-depleted RNA libraries for five pollen developmental stages (pollen mother cells, tetrad, uninucleate pollen, binucleate pollen, and mature pollen) were constructed. A total of about 23 gigabytes of high-quality clean data with more than 233 million reads was obtained (Table S2). After analyzing the sequencing data with three circRNA identification tools, more than 2000 circRNAs were obtained (Table S3). Out of the total number of circRNAs, we identified 1180 unique circRNAs in all five pollen developmental stages with 824 circRNAs for find_circ, 380 circRNAs for CIRI2, and 137 circRNAs for CIRCExplorer2 (Figure S2 and Table S4). When comparing the number of circRNAs found by all three tools, only six circRNAs were common between all, and more circRNAs were common between CIRI2 and find_circ (Figure S2). Among 1180 identified circRNAs, 1034 (87.63%) circRNAs were predicted to have at least one exon from protein-coding genes (Figure 2A), 85 (7.20%) circRNAs were generated from intergenic regions, and of the remaining circRNAs, 61 (5.17%) were intronic. While 133 genes could produce two or more types of circRNAs through alternative back-splicing, 737 genes produced only one circRNA isoform (Figure 2B). Moreover, the parent genes of circRNAs were unevenly distributed on different chromosomes; chromosome nine with 184 circRNAs and chromosome four with 63 circRNAs produced the most and the least circRNAs, respectively, and 21 circRNAs were produced from four scaffolds (Figure 2C). The length distribution of circRNAs was mainly distributed between 100 to 600 bp (Figure 2D). Finally, we visualized circRNA expression vs. pollen developmental stages using a clustering heatmap (Figure 3).

### 3.2. The Expression Patterns of circRNAs in B. rapa Pollen Development

**Figure 4:**
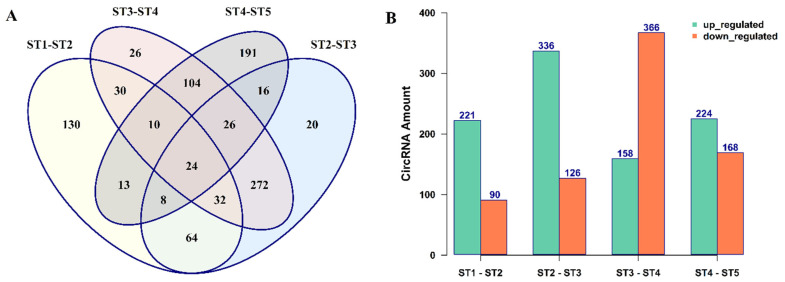
Differential expression patterns of circRNAs during pollen development in *B. rapa*. (**A**) A Venn diagram showing the number of differentially expressed circRNAs during pollen developmental stages. (**B**) Histograms represent the regulation of differentially expressed circRNAs during pollen developmental stages. ST1: stage 1 (pollen mother cells), ST2: stage 2 (tetrad), ST3: stage 3 (uninucleate pollen), ST4: stage 4 (binucleate pollen), and ST5: stage 5 (mature pollen).

To investigate whether the circRNAs are expressed in a specific manner during *B. rapa* pollen development, we compared the expression patterns of circRNAs in each pollen developmental stage with its previous stage (Table S5). The results show that 966 circRNAs had a significant differential expression, of which 367 circRNAs displayed stage-specific expression patterns. Comparing the expression of circRNAs between stages, a more distinct expression was observed when pollen developed from binucleate pollen to mature pollen or developed from pollen mother cell to tetrad. (Figure 4A). Additionally, more circRNAs tended to up-regulate during pollen development, except when pollen developed from uninucleate pollen to binucleate pollen, where more circRNAs were down-regulated (Figure 4B). Finally, we visualized the expression profile of the 60 most significant (α ≥ 0.001) differential expressed circRNAs (Tables S5 and S6) among different stages of pollen development (Figure S3). The expression analysis results indicate that circRNAs have a distinctive expression pattern during pollen development, pointing towards their defined stage-specific roles in pollen developmental progression.

### 3.3. Conservation Analysis of circRNAs between B. rapa and other Plant Species

The comparison of identified circRNAs in this study with the collection of circRNAs obtained from PlantcircBase showed that 38.71% of *B. rapa* circRNAs were homologous to the circRNAs in the database of which more than 35% were homologous to the *Arabidopsis* circRNAs (Table 1).

### 3.4. Functional Annotation Analysis of CircRNAs Parent Genes

**Figure 5:**
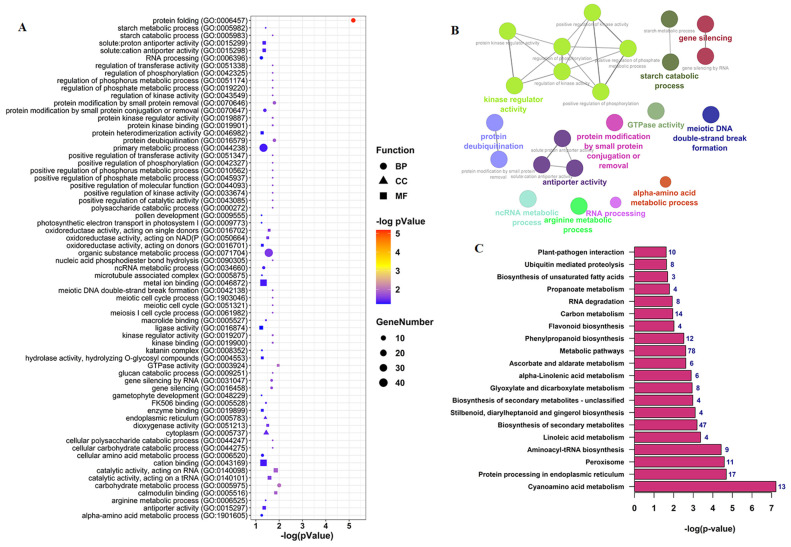
Functional analysis of circRNA parent genes during *B. rapa* pollen development. (**A**) GO enrichment analysis of the host genes of differentially expressed circRNAs. (**B**) ClueGO network visualizing the most significant GO terms for circRNA parent genes. Colors represent GO groups. (**C**) KEGG enrichment analysis of the host genes of differentially expressed circRNAs. The number on the bars shows the number of genes enriched in the corresponding pathways.

To explore the potential functions of circRNAs during pollen development, we performed GO enrichment analysis on the parental genes of all the differential expressed circRNAs. The results show that circRNA parent genes belonged to three GO categories: biological process, molecular function, and cellular component (Table S7). For the biological process, the circRNA–host genes were enriched in a variety of metabolic, catabolic, and cell processes such as protein folding, glucan catabolic process, regulation of phosphorus metabolism, meiotic DNA double-strand break formation process, meiotic cell cycle process, meiosis I cell cycle process, and ncRNA metabolic process. In the molecular function, the enriched GO terms included GTPase activity, calmodulin binding, catalytic activity acting on RNA, kinase regulator activity, and metal ion binding. In the cellular component category, only four terms, cytoplasm, endoplasmic reticulum, microtubule-associated complex, and katanin complex, were enriched (Figure 5A,B).

To further investigate the function of circRNA host genes, Encyclopedia of Genes and Genomes (KEGG) pathway enrichment analysis was conducted. The results reveal the enrichment of 23 significant pathways (Table S8), including cyanoamino acid metabolism, protein processing in the endoplasmic reticulum, peroxisome, and aminoacyl-tRNA biosynthesis, among others (Figure 5C).

### 3.5. Prediction of miRNA Target Sites in circRNAs

**Figure 6:**
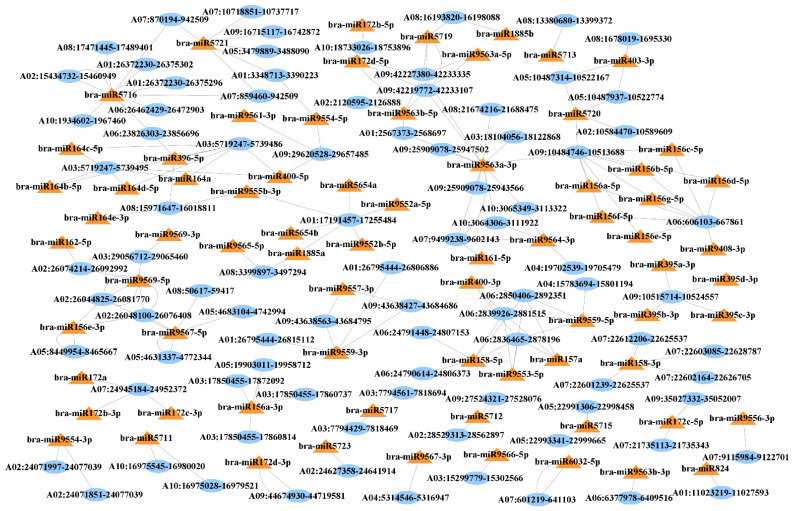
CircRNA–miRNA interaction network for differentially expressed circRNAs during pollen development in *B. rapa*. Blue ovals: circRNA IDs, orange triangles: miRNA IDs.

To further evaluate the function of circRNAs in the post-transcriptional regulation of genes in pollen development, we predicted the binding sites of miRNAs in the sequences of differential expressed circRNAs (Table S9). The results show that 88 circRNAs contained putative miRNA-binding sites for 73 miRNAs. Of these 88 circRNAs, 49 (55.68%) had only one miRNA-binding site, followed by 22 circRNAs (25%) with two miRNA-binding sites, and the remaining circRNAs (19.32%) contained binding sites for three to nine miRNAs (Figure 6). For example, circRNA A06:606103-667861 and A09:10484746-10513688 had binding sites for eight and nine miRNAs, respectively. MiRNAs could be targeted by one or more circRNAs as well. Among 74 identified miRNAs, 27 (36.49%) were targeted by a single circRNA, 24 miRNAs (32.43%) were targeted by two circRNAs, and the remaining miRNAs (31.08%) were targeted by three to twelve circRNAs (Figure 6). For instance, bra-miR9569-5p, bra-miR5716, and bra-miR9563a-3p were targeted by seven, ten, and twelve circRNAs, respectively.

**Figure 7:**
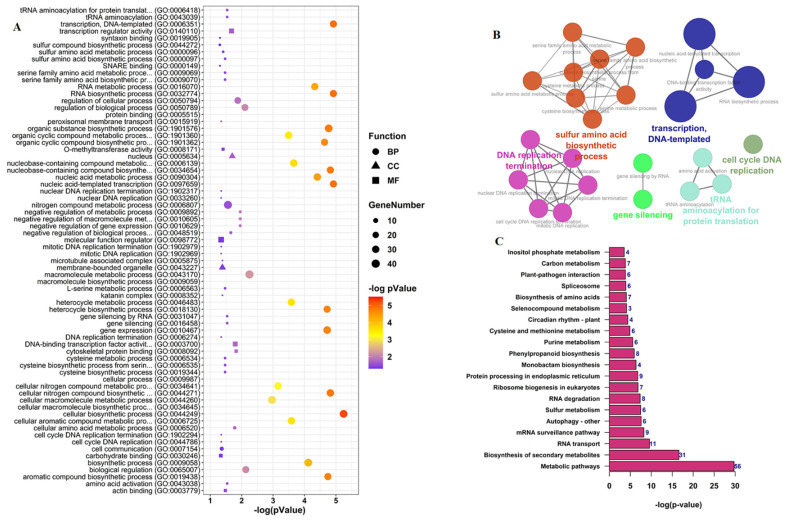
Functional analysis of miRNA target genes during *B. rapa* pollen development. (**A**) GO enrichment analysis. (**B**) ClueGO network visualizing the most significant GO terms—colors represent GO groups. (**C**) KEGG enrichment analysis. The number on the bars represent the number of genes enriched in the corresponding pathways.

We also predicted miRNA targets and performed functional enrichment analysis to investigate what processes in the cell could be affected by the interaction of circRNAs and miRNAs. The results show that the 73 predicted miRNAs could interact with 741 genes (Table S10) belong to various GO terms, mainly DNA processes and replication, amino acid metabolic processes, and gene expression processes (Table S10, Figure 7A,B). KEGG pathway enrichment analysis showed that miRNA target genes are involved in multiple pathways such as metabolic pathways, biosynthesis of secondary metabolites, and RNA transport, among others (Table S11, Figure 7C).

### 3.6. Validation of circRNAs Using Divergent Primers

**Figure 8:**
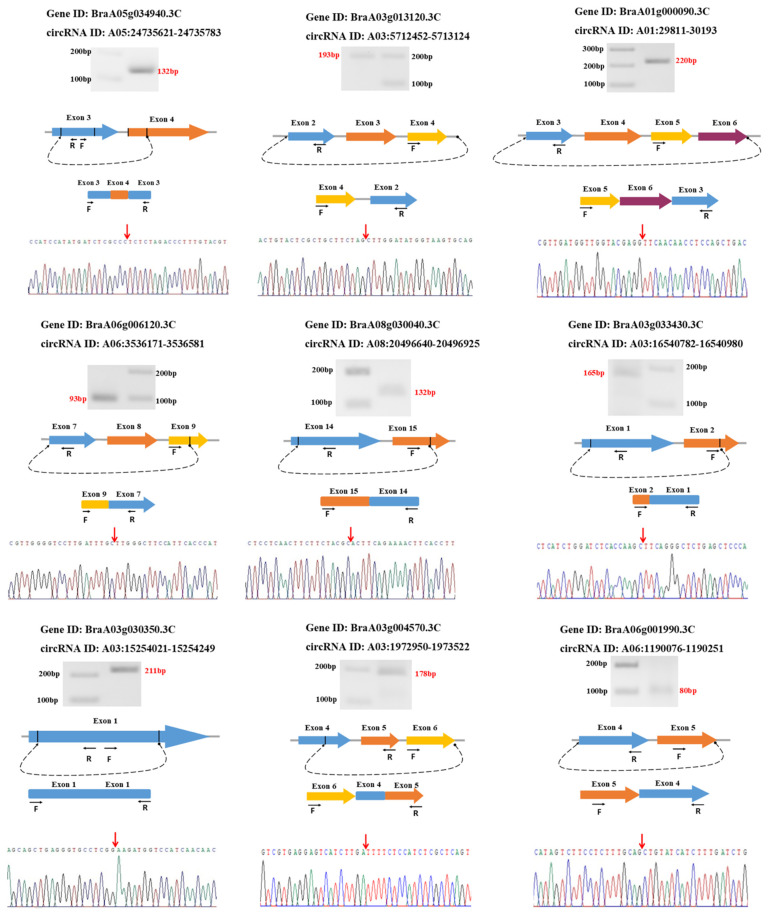
CircRNA validation using divergent primers and Sanger sequencing. From the top: the name of the circRNA parent gene, circRNA ID, the amplified band using divergent primers and cDNA, schematic representation of the genomic region of selected circRNAs and their amplified junction, dashed curved arrows represent head-to-tail back-splicing points, the two sets of arrows indicate the position of divergent primers on corresponding exons, conjunction validation using Sanger sequencing, and red arrows show the position of the junction point.

To confirm the accuracy of the identified circRNAs, we selected 15 circRNAs for experimental validation using PCR and Sanger sequencing. Using a set of divergent primers designed for each circRNA, we successfully amplified nine circRNAs using nine pairs of divergent primers. Then, all the amplified PCR products were further validated by sequencing to confirm the presence of the back-spliced junctions (Figure 8). Our validated circRNAs are composed of one to three exons, except for circRNA A03:5712452-5713124, which has an intron in its conjunction site.

## 4. Discussion

There is increasing evidence that circular RNAs are not simply rare splicing events but are highly regulated molecules with unique selected sequences from different genomic regions [54]. In animals, circRNAs can carry out important biological functions in a cell-type, tissue-, or developmental-stage-specific manner [55]. Although most of the circRNAs are expressed at a low level compared with their cognate mRNAs, they can play important roles because they are naturally resistant to diverse exonucleases, and their half-life is much longer than mRNAs [54,56]. To date, thousands of distinct circRNAs have been found in several taxa, including different species of plants [57,58]. Various studies have suggested that plant circRNAs are closely associated with growth and development [59,60,61]. In the present study, we investigated the expression and potential function of circRNAs during pollen development in *B. rapa.* According to the comparison studies, circRNA detection tools have been designed with different detection algorithms, and no single method provides all the metrics needed for circRNA identification [36,62,63,64]. Here, we used CIRI2, find_circ, and CIRCExplorer2, the top three identification tools for circRNA analysis in the literature [36], to achieve a better overview of the circRNA expression profile in our datasets. After combining the results, only six identified circRNAs were common between the three tools, which is normal as each tool uses a different aligner and metrics for identifying circRNAs in a given dataset [64]. Different tools, especially when compared pairwise, may share a subset of identified circRNAs, but in most cases, this number decreases after combining the results from more than two tools [64]. In total, we identified 1180 circRNAs in all five stages of pollen development. Compared with previous reports, this number is similar to other plant species such as *Arabidopsis* (970) [65], *Gossypium arboretum* (1041), and *Gossypium raimondii* (1478) [66]. We found that during pollen development, circRNAs originated from different genomic regions, but most of our identified circRNAs (87.63%) were generated from exonic regions, which is consistent with previous studies on many plant species such as *Arabidopsis* (85.1%) [67], Chinese cabbage (70.49%) [68], and *Brassica campestris* (63.13%) [30]. Most of the genes can produce only one isoform of circRNAs; however, multiple isoforms of circRNAs produced through alternative back-splicing from a single gene have also been observed [69,70,71]. Here, we found that most of the circRNA parent genes (about 83%) generate only one circRNA isoform, with the remaining 17% of the genes able to produce two or more circRNA isoforms. Conservation between different plant species is another feature of circRNAs that has been shown in multiple studies [67,72,73,74]. Although more than 37% of our identified circRNAs were found to be conserved between *B. rapa* and other plant species, the majority of them were conserved between *B. rapa* and *Arabidopsis*, the two species from the same family with closely related genomes [75]. When Arabidopsis was not taken into account, only about 3% of *B. rapa* circRNAs were conserved with circRNAs from other plant species. The low conservation rate could be due to the differences between datasets, as they were prepared from various tissues and/or under stress conditions, or the various bioinformatics tools that have been used for analyzing those datasets [73].

The expression of circRNAs changes during different stages of growth and development. For instance, by analyzing the expression profile of circRNAs in various plant species, it was revealed that circRNAs are closely involved in leaf growth in *Arabidopsis* [59], fruit ripening in pepper [60], and pollen development in *B. campestris* [30], rice [28], and soybean [29]. Herein, our differential expression analysis revealed that the stage-specific expression pattern of circRNAs changes during pollen development. We found 130 differentially expressed circRNAs while pollen mother cells developed to tetrad, and 191 differentially expressed circRNAs while binucleate pollen developed to mature pollen. We noted that the most common circRNAs were shared between two closely related stages. For example, 272 shared differentially expressed circRNAs when tetrads developed to microspores and then to binucleate pollens. These findings indicate that circRNA expression is related to different pollen developmental stages, and they have the potential to play important roles during pollen development in *B. rapa*.

Previous studies showed that circRNAs could regulate various processes such as chromatin structure, gene expression, translation, and even cell division [76,77,78]. For example, centromeric-retrotransposon-derived circRNAs in maize can bind to centromeres via R-loops to promote chromatin looping in centromere regions [76]. Exon–intron-containing circRNAs in human cells can increase the transcription of their parental genes by interacting with U1 small nuclear ribonucleoproteins at the promoters [77], or in *Arabidopsis*, circRNAs can form a strong RNA–DNA hybrid, resulting in transcriptional pausing [78]. CircRNAs also can form circRNA–protein structures and affect translation and cell cycle regulation. One such example is CircPABPN1, which can suppress HuR from binding to its cognate mRNA, resulting in reduced translation of PABPN1 mRNA [79]. Another example is circFOXO3, which has multiple binding sites for cell-cycle-regulating proteins such as p53, CDK-2, and p21. It was reported that circFOXO3, p21, and CDK-2 could form a ternary structure and inhibit CDK-2/Cyclin-E complex activation, which is necessary for G1 to S phase transition [80]. During pollen development, several mitotic and meiotic cell divisions occur, which involve the expression of a few thousand genes [16,81]. Studies showed that during pollen development, the tapetum endoplasmic reticulum was highly involved in biosynthesis, folding, and secreting proteins [82,83]. For pollen wall formation, lipidic components and polysaccharides are required as tapetum secretes lipid components onto the pollen surface [84,85], or β-glucosidase, which is involved in the regulation of polysaccharide metabolism, downregulated in the sterile floral buds of *B. rapa* [86]. As circRNAs’ functions may be related to their parent gene’s function, we annotated the biological roles of circRNAs’ parent genes using GO and KEGG analysis to better understand the potential function of circRNAs in pollen development. We noted that circRNA parent genes could be involved in various important processes and functions related to pollen development such as protein synthesis and fate, cell cycle and DNA processing, kinase and phosphorus activities, polysaccharide metabolism, gene silencing, RNA processing, and antiporter activities. KEGG analysis showed that the circRNAs’ parent genes are involved in 23 pathways, mainly amino acid metabolism and protein processing, lipid biosynthesis and metabolism, metabolic pathways, carbon metabolism, and RNA degradation. Similar results were observed when the function of miRNA target genes was investigated. Based on our results and previous findings, we can assume that circRNAs might play vital functions during pollen development in *B. rapa*.

Small RNAs such as miRNAs can regulate gene expression through mRNA cleavage, translational repression, or gene silencing via miRNA-directed DNA methylation [87]. Studies on animal and human cells have reported that circRNAs can bind to miRNAs and sequester them from their target mRNAs to regulate gene expression at the post-transcriptional level [88]. For example, it has been shown that *circSry,* associated with testis development in mice, contains 16 binding sites for miR-138 [89], and *CDR1as*, which is a highly expressed circRNA in mammalian brains, contains more than 70 binding sites for miR-7. Studies showed that *CDR1as* regulates gene expression by acting as miR-7 storage (sponge) and ensuring the release of an appropriate amount of miR-7 to the target mRNAs [8,90,91]. Prediction of circRNA–miRNA networks in plant species revealed that plant circRNAs, compared with animals and humans, have fewer interactions with miRNAs, suggesting that the main function of plant circRNAs might not be an miRNA decoy [92]. Nevertheless, several studies proposed that plant circRNAs could target miRNAs by acting as competing endogenous RNAs in post-transcriptional regulation of the genes [28,29,30,93,94]. For example, circRNAs were proposed to function as miRNA sponges in flower development in *Arabidopsis* [93], anther development in *B. campestris* [30], and pollen development in rice and soybean [28,29]. A database named “GreenCircRNA” collected all the identified circRNAs in different plant species and predicted circRNA–miRNAs interactions for each species [95]. In the present study, we found 88 circRNAs with putative miRNA binding sites. A few of them had multiple binding sites for the same or different miRNAs, suggesting that circRNAs could interact with miRNAs or act as miRNA sponges to regulate pollen development. We also noted some well-known and important miRNAs in our predicted network, such as miR156, miR157, miR158, miR161, miR162, miR164, miR172, miR395, and miR396. These miRNAs proved to be involved in many biological and developmental processes such as vegetative growth, flowering, fertility, and fruit ripening [87,96,97,98]. For example, we found nine circRNAs with binding sites for miR156, seven circRNA with binding sites for miR172, four circRNAs with binding sites for miR396, and three circRNAs with binding sites for miR164, which all are known to regulate flower development in *Arabidopsis* [99,100], barley [101], tomato [102], *Brassica napus* [103], and strawberry [104]. In addition, we found nine circRNAs with binding sites for miR158 that were previously reported as key regulatory miRNA during pollen development in *Brassica campestris* [105]. Accordingly, our results suggest that circRNAs could act as ceRNAs during pollen development in *B. rapa*.

## 5. Conclusions

In conclusion, by studying the profile of circRNA expression during pollen development in *B. rapa*, we identified 1180 circRNAs, of which 367 circRNAs showed stage-specific expression. Functional characterization of circRNA host genes revealed that circRNAs were mainly related to biological and molecular processes in pollen development such as mitosis and meiosis cell cycles, protein biosynthesis, protein modification, and polysaccharide processes. Moreover, 88 circRNAs were found to contain miRNA-binding sites, suggesting the role of circRNAs in gene expression as post-transcriptional regulatory elements. Our study revealed the potential functions of circRNAs during pollen development and paves the way for further experiments on studying the molecular mechanism of these new regulatory RNA molecules.

## Figures and Tables

**Table 1:** Summary of conserved circRNAs between *B. rapa* and other plant species.

Plant Species	No. of CircRNAs	No. of Conserved CircRNAs	% Conservation
*Arabidopsis thaliana*	52,393	421/1180	35.68
*Oryza sativa*	40,311	9/1180	0.76
*Glycine max*	8148	8/1180	0.68
*Gossypium hirsutum*	3944	5/1180	0.42
*Cucumis sativus*	4832	4/1180	0.34
*Solanum tuberosum*	1728	3/1180	0.25
*Solanum lycopersicum*	3727	3/1180	0.25
*Zea mays*	6434	3/1180	0.25
*Gossypium raimondii*	1478	1/1180	0.08

## Data Availability

All data generated in this study are available in the article and its Supplementary Materials.

## Supplementary Materials

The following are available online at https://www.mdpi.com/article/10.3390/ijms221910297/s1.

## References

[1] De Klerk E., AC‘t Hoen P. (2015). Alternative mRNA transcription, processing, and translation: Insights from RNA sequencing. Trends Genet..

[2] Mattick J.S., Makunin I.V. (2006). Non-coding RNA. Hum. Mol. Genet..

[3] Cech T.R., Steitz J.A. (2014). The noncoding RNA revolution—Trashing old rules to forge new ones. Cell.

[4] Kaikkonen M.U., Lam M.T., Glass C.K. (2011). Non-coding RNAs as regulators of gene expression and epigenetics. Cardiovasc. Res..

[5] Meng X., Li X., Zhang P., Wang J., Zhou Y., Chen M. (2017). Circular RNA: An emerging key player in RNA world. Brief. Bioinform..

[6] Sinha T., Panigrahi C., Das D., Chandra Panda A. (2021). Circular RNA translation, a path to hidden proteome. Wiley Interdiscip. Rev. RNA.

[7] Zhou M., Xiao M.-S., Li Z., Huang C. (2020). New progresses of circular RNA biology: From nuclear export to degradation. RNA Biol..

[8] Xiao M.-S., Ai Y., Wilusz J.E. (2020). Biogenesis and functions of circular RNAs come into focus. Trends Cell Biol..

[9] Zhang P., Li S., Chen M. (2020). Characterization and function of circular RNAs in plants. Front. Mol. Biosci..

[10] Guria A., Sharma P., Natesan S., Pandi G. (2020). Circular RNAs—The road less traveled. Front. Mol. Biosci..

[11] Wang P.L., Bao Y., Yee M.-C., Barrett S.P., Hogan G.J., Olsen M.N., Dinneny J.R., Brown P.O., Salzman J. (2014). Circular RNA is expressed across the eukaryotic tree of life. PLoS ONE.

[12] Golicz A.A., Bhalla P.L., Singh M.B. (2018). lncRNAs in plant and animal sexual reproduction. Trends Plant Sci..

[13] Heo J.B., Sung S. (2011). Vernalization-mediated epigenetic silencing by a long intronic noncoding RNA. Science.

[14] Csorba T., Questa J.I., Sun Q., Dean C. (2014). Antisense COOLAIR mediates the coordinated switching of chromatin states at FLC during vernalization. Proc. Natl. Acad. Sci. USA.

[15] Komiya R., Ohyanagi H., Niihama M., Watanabe T., Nakano M., Kurata N., Nonomura K.I. (2014). Rice germline-specific A rgonaute MEL 1 protein binds to phasi RNA s generated from more than 700 linc RNA s. Plant J..

[16] Singh M.B., Bhalla P.L. (2007). Control of male germ-cell development in flowering plants. Bioessays.

[17] Haerizadeh F., Wong C.E., Bhalla P.L., Gresshoff P.M., Singh M.B. (2009). Genomic expression profiling of mature soybean (Glycine max) pollen. BMC Plant Biol..

[18] Russell S.D., Gou X., Wong C.E., Wang X., Yuan T., Wei X., Bhalla P.L., Singh M.B. (2012). Genomic profiling of rice sperm cell transcripts reveals conserved and distinct elements in the flowering plant male germ lineage. New Phytol..

[19] Golicz A.A., Allu A.D., Li W., Lohani N., Singh M.B., Bhalla P.L. (2021). A dynamic intron retention program regulates the expression of several hundred genes during pollen meiosis. Plant Reprod..

[20] Janousek B., Zluvova J., Vyskot B. (2000). Histone H4 acetylation and DNA methylation dynamics during pollen development. Protoplasma.

[21] Sharma N., Russell S.D., Bhalla P.L., Singh M.B. (2011). Putative cis-regulatory elements in genes highly expressed in rice sperm cells. BMC Res. Notes.

[22] Okada T., Singh M.B., Bhalla P.L. (2006). Histone H3 variants in male gametic cells of lily and H3 methylation in mature pollen. Plant Mol. Biol..

[23] Singh M., Bhalla P.L., Xu H., Singh M.B. (2003). Isolation and characterization of a flowering plant male gametic cell-specific promoter. FEBS Lett..

[24] Xu H., Swoboda I., Bhalla P.L., Singh M.B. (1999). Male gametic cell-specific expression of H2A and H3 histone genes. Plant Mol. Biol..

[25] Ma J., Yan B., Qu Y., Qin F., Yang Y., Hao X., Yu J., Zhao Q., Zhu D., Ao G. (2008). Zm401, a short-open reading-frame mRNA or noncoding RNA, is essential for tapetum and microspore development and can regulate the floret formation in maize. J. Cell. Biochem..

[26] Ding J., Lu Q., Ouyang Y., Mao H., Zhang P., Yao J., Xu C., Li X., Xiao J., Zhang Q. (2012). A long noncoding RNA regulates photoperiod-sensitive male sterility, an essential component of hybrid rice. Proc. Natl. Acad. Sci. USA.

[27] Huang L., Dong H., Zhou D., Li M., Liu Y., Zhang F., Feng Y., Yu D., Lin S., Cao J. (2018). Systematic identification of long non-coding RNA s during pollen development and fertilization in Brassica rapa. Plant J..

[28] Wang Y., Xiong Z., Li Q., Sun Y., Jin J., Chen H., Zou Y., Huang X., Ding Y. (2019). Circular RNA profiling of the rice photo-thermosensitive genic male sterile line Wuxiang S reveals circRNA involved in the fertility transition. BMC Plant Biol..

[29] Chen L., Ding X., Zhang H., He T., Li Y., Wang T., Li X., Jin L., Song Q., Yang S. (2018). Comparative analysis of circular RNAs between soybean cytoplasmic male-sterile line NJCMS1A and its maintainer NJCMS1B by high-throughput sequencing. BMC Genom..

[30] Liang Y., Zhang Y., Xu L., Zhou D., Jin Z., Zhou H., Lin S., Cao J., Huang L. (2019). CircRNA Expression Pattern and ceRNA and miRNA–mRNA Networks Involved in Anther Development in the CMS Line of Brassica campestris. Int. J. Mol. Sci..

[31] Lohani N., Singh M.B., Bhalla P. (2020). RNA-seq highlights molecular events associated with impaired pollen-pistil interactions following short-term heat stress in Brassica napus. Front. Plant Sci..

[32] Wang X., Wang H., Wang J., Sun R., Wu J., Liu S., Bai Y., Mun J.-H., Bancroft I., Cheng F. (2011). The genome of the mesopolyploid crop species Brassica rapa. Nat. Genet..

[33] Li H., Durbin R. (2009). Fast and accurate short read alignment with Burrows–Wheeler transform. Bioinformatics.

[34] Langmead B., Salzberg S.L. (2012). Fast gapped-read alignment with Bowtie 2. Nat. Methods.

[35] Dobin A., Davis C.A., Schlesinger F., Drenkow J., Zaleski C., Jha S., Batut P., Chaisson M., Gingeras T.R. (2013). STAR: Ultrafast universal RNA-seq aligner. Bioinformatics.

[36] Chen L., Wang C., Sun H., Wang J., Liang Y., Wang Y., Wong G. (2021). The bioinformatics toolbox for circRNA discovery and analysis. Brief. Bioinform..

[37] Gao Y., Zhang J., Zhao F. (2018). Circular RNA identification based on multiple seed matching. Brief. Bioinform..

[38] Memczak S., Jens M., Elefsinioti A., Torti F., Krueger J., Rybak A., Maier L., Mackowiak S.D., Gregersen L.H., Munschauer M. (2013). Circular RNAs are a large class of animal RNAs with regulatory potency. Nature.

[39] Zhang X.-O., Dong R., Zhang Y., Zhang J.-L., Luo Z., Zhang J., Chen L.-L., Yang L. (2016). Diverse alternative back-splicing and alternative splicing landscape of circular RNAs. Genome Res..

[40] Quinlan A.R., Hall I.M. (2010). BEDTools: A flexible suite of utilities for comparing genomic features. Bioinformatics.

[41] Tarazona S., García-Alcalde F., Dopazo J., Ferrer A., Conesa A. (2011). Differential expression in RNA-seq: A matter of depth. Genome Res..

[42] Robinson M.D., Oshlack A. (2010). A scaling normalization method for differential expression analysis of RNA-seq data. Genome Biol..

[43] R Foundation for Statistical Computing (2013). R: A Language and Environment for Statistical Computing.

[44] Wickham H. (2016). ggplot2: Elegant Graphics for Data Analysis.

[45] Warnes G.R., Bolker B., Bonebakker L., Gentleman R., Huber W., Liaw A., Lumley T., Maechler M., Magnusson A., Moeller S. (2020). gplots: Various R Programming Tools for Plotting Data. R Package Vers 3.1.1..

[46] Altschul S.F., Gish W., Miller W., Myers E.W., Lipman D.J. (1990). Basic local alignment search tool. J. Mol. Biol..

[47] Alexa A., Rahnenfuhrer J. (2020). topGO: Enrichment Analysis for Gene Ontology. R Package Vers 2.42.0.

[48] Xie C., Mao X., Huang J., Ding Y., Wu J., Dong S., Kong L., Gao G., Li C.-Y., Wei L. (2011). KOBAS 2.0: A web server for annotation and identification of enriched pathways and diseases. Nucleic Acids Res..

[49] Bindea G., Mlecnik B., Hackl H., Charoentong P., Tosolini M., Kirilovsky A., Fridman W.-H., Pagès F., Trajanoski Z., Galon J. (2009). ClueGO: A Cytoscape plug-in to decipher functionally grouped gene ontology and pathway annotation networks. Bioinformatics.

[50] Dai X., Zhao P.X. (2011). psRNATarget: A plant small RNA target analysis server. Nucleic Acids Res..

[51] Shannon P., Markiel A., Ozier O., Baliga N.S., Wang J.T., Ramage D., Amin N., Schwikowski B., Ideker T. (2003). Cytoscape: A software environment for integrated models of biomolecular interaction networks. Genome Res..

[52] Fahlgren N., Howell M.D., Kasschau K.D., Chapman E.J., Sullivan C.M., Cumbie J.S., Givan S.A., Law T.F., Grant S.R., Dangl J.L. (2007). High-throughput sequencing of Arabidopsis microRNAs: Evidence for frequent birth and death of MIRNA genes. PLoS ONE.

[53] Untergasser A., Cutcutache I., Koressaar T., Ye J., Faircloth B.C., Remm M., Rozen S.G. (2012). Primer3—New capabilities and interfaces. Nucleic Acids Res..

[54] Wilusz J.E. (2018). A 360 view of circular RNAs: From biogenesis to functions. Wiley Interdiscip. Rev. RNA.

[55] Ebbesen K.K., Kjems J., Hansen T.B. (2016). Circular RNAs: Identification, biogenesis and function. Biochim. Biophys. Acta Gene Regul. Mech..

[56] Lasda E., Parker R. (2014). Circular RNAs: Diversity of form and function. RNA.

[57] Glažar P., Papavasileiou P., Rajewsky N. (2014). circBase: A database for circular RNAs. RNA.

[58] Chu Q., Zhang X., Zhu X., Liu C., Mao L., Ye C., Zhu Q.-H., Fan L. (2017). PlantcircBase: A database for plant circular RNAs. Mol. Plant.

[59] Liu T., Zhang L., Chen G., Shi T. (2017). Identifying and characterizing the circular RNAs during the lifespan of Arabidopsis leaves. Front. Plant Sci..

[60] Yang S., Yang T., Tang Y., Aisimutuola P., Zhang G., Wang B., Li N., Wang J., Yu Q. (2020). Transcriptomic profile analysis of non-coding RNAs involved in Capsicum chinense Jacq. fruit ripening. Sci. Hortic..

[61] Cheng J., Zhang Y., Li Z., Wang T., Zhang X., Zheng B. (2018). A lariat-derived circular RNA is required for plant development in Arabidopsis. Sci. China Life Sci..

[62] Zeng X., Lin W., Guo M., Zou Q. (2017). A comprehensive overview and evaluation of circular RNA detection tools. PLoS Comput. Biol..

[63] Jakobi T., Dieterich C. (2019). Computational approaches for circular RNA analysis. Wiley Interdiscip. Rev. RNA.

[64] Hansen T.B. (2018). Improved circRNA identification by combining prediction algorithms. Front. Cell Dev. Biol..

[65] Sun X., Wang L., Ding J., Wang Y., Wang J., Zhang X., Che Y., Liu Z., Zhang X., Ye J. (2016). Integrative analysis of Arabidopsis thaliana transcriptomics reveals intuitive splicing mechanism for circular RNA. FEBS Lett..

[66] Zhao T., Wang L., Li S., Xu M., Guan X., Zhou B. (2017). Characterization of conserved circular RNA in polyploid Gossypium species and their ancestors. FEBS Lett..

[67] Chen G., Cui J., Wang L., Zhu Y., Lu Z., Jin B. (2017). Genome-wide identification of circular RNAs in Arabidopsis thaliana. Front. Plant Sci..

[68] Wang W., Wang J., Wei Q., Li B., Zhong X., Hu T., Hu H., Bao C. (2019). Transcriptome-wide identification and characterization of circular RNAs in leaves of Chinese cabbage (*Brassica rapa* L. ssp. pekinensis) in response to calcium deficiency-induced tip-burn. Sci. Rep..

[69] Zhang X., Ma X., Ning L., Li Z., Zhao K., Li K., He J., Yin D. (2019). Genome-wide identification of circular RNAs in peanut (*Arachis hypogaea* L.). BMC Genom..

[70] Lu T., Cui L., Zhou Y., Zhu C., Fan D., Gong H., Zhao Q., Zhou C., Zhao Y., Lu D. (2015). Transcriptome-wide investigation of circular RNAs in rice. RNA.

[71] Lv L., Yu K., Lü H., Zhang X., Liu X., Sun C., Xu H., Zhang J., He X., Zhang D. (2020). Transcriptome-wide identification of novel circular RNAs in soybean in response to low-phosphorus stress. PLoS ONE.

[72] Ye C.Y., Chen L., Liu C., Zhu Q.H., Fan L. (2015). Widespread noncoding circular RNA s in plants. New Phytol..

[73] Gao Z., Li J., Luo M., Li H., Chen Q., Wang L., Song S., Zhao L., Xu W., Zhang C. (2019). Characterization and cloning of grape circular RNAs identified the cold resistance-related Vv-circATS1. Plant Physiol..

[74] Tong W., Yu J., Hou Y., Li F., Zhou Q., Wei C., Bennetzen J.L. (2018). Circular RNA architecture and differentiation during leaf bud to young leaf development in tea (*Camellia sinensis*). Planta.

[75] Rutter M.T., Cross K.V., Van Woert P.A. (2012). Birth, death and subfunctionalization in the Arabidopsis genome. Trends Plant Sci..

[76] Liu Y., Su H., Zhang J., Liu Y., Feng C., Han F. (2020). Back-spliced RNA from retrotransposon binds to centromere and regulates centromeric chromatin loops in maize. PLoS Biol..

[77] Li Z., Huang C., Bao C., Chen L., Lin M., Wang X., Zhong G., Yu B., Hu W., Dai L. (2015). Exon-intron circular RNAs regulate transcription in the nucleus. Nat. Struct. Mol. Biol..

[78] Conn V.M., Hugouvieux V., Nayak A., Conos S.A., Capovilla G., Cildir G., Jourdain A., Tergaonkar V., Schmid M., Zubieta C. (2017). A circRNA from SEPALLATA3 regulates splicing of its cognate mRNA through R-loop formation. Nat. Plants.

[79] Abdelmohsen K., Panda A.C., Munk R., Grammatikakis I., Dudekula D.B., De S., Kim J., Noh J.H., Kim K.M., Martindale J.L. (2017). Identification of HuR target circular RNAs uncovers suppression of PABPN1 translation by CircPABPN1. RNA Biol..

[80] Du W.W., Yang W., Liu E., Yang Z., Dhaliwal P., Yang B.B. (2016). Foxo3 circular RNA retards cell cycle progression via forming ternary complexes with p21 and CDK2. Nucleic Acids Res..

[81] Rutley N., Twell D. (2015). A decade of pollen transcriptomics. Plant Reprod..

[82] Fragkostefanakis S., Mesihovic A., Hu Y., Schleiff E. (2016). Unfolded protein response in pollen development and heat stress tolerance. Plant Reprod..

[83] Singh M.B., Lohani N., Bhalla P.L. (2021). The Role of Endoplasmic Reticulum Stress Response in Pollen Development and Heat Stress Tolerance. Front. Plant Sci..

[84] Jiang J., Zhang Z., Cao J. (2013). Pollen wall development: The associated enzymes and metabolic pathways. Plant Biol..

[85] Sharma A., Singh M.B., Bhalla P.L. (2015). Ultrastructure of microsporogenesis and microgametogenesis in Brachypodium distachyon. Protoplasma.

[86] Dong X., Feng H., Xu M., Lee J., Kim Y.K., Lim Y.P., Piao Z., Park Y.D., Ma H., Hur Y. (2013). Comprehensive analysis of genic male sterility-related genes in *Brassica rapa* using a newly developed Br300K oligomeric chip. PLoS ONE.

[87] Waititu J.K., Zhang C., Liu J., Wang H. (2020). Plant Non-Coding RNAs: Origin, Biogenesis, Mode of Action and Their Roles in Abiotic Stress. Int. J. Mol. Sci..

[88] Li X., Yang L., Chen L.-L. (2018). The biogenesis, functions, and challenges of circular RNAs. Mol. Cell.

[89] Hansen T.B., Jensen T.I., Clausen B.H., Bramsen J.B., Finsen B., Damgaard C.K., Kjems J. (2013). Natural RNA circles function as efficient microRNA sponges. Nature.

[90] Kleaveland B., Shi C.Y., Stefano J., Bartel D.P. (2018). A network of noncoding regulatory RNAs acts in the mammalian brain. Cell.

[91] Piwecka M., Glažar P., Hernandez-Miranda L.R., Memczak S., Wolf S.A., Rybak-Wolf A., Filipchyk A., Klironomos F., Jara C.A.C., Fenske P. (2017). Loss of a mammalian circular RNA locus causes miRNA deregulation and affects brain function. Science.

[92] Chu Q., Bai P., Zhu X., Zhang X., Mao L., Zhu Q.-H., Fan L., Ye C.-Y. (2020). Characteristics of plant circular RNAs. Brief. Bioinform..

[93] Frydrych Capelari É., da Fonseca G.C., Guzman F., Margis R. (2019). Circular and micro RNAs from Arabidopsis thaliana flowers are simultaneously isolated from AGO-IP libraries. Plants.

[94] Liu S., Wu L., Qi H., Xu M. (2019). LncRNA/circRNA–miRNA–mRNA networks regulate the development of root and shoot meristems of Populus. Ind. Crop. Prod..

[95] Zhang J., Hao Z., Yin S., Li G. (2020). GreenCircRNA: A database for plant circRNAs that act as miRNA decoys. Database.

[96] Luján-Soto E., Dinkova T.D. (2021). Time to Wake Up: Epigenetic and Small-RNA-Mediated Regulation during Seed Germination. Plants.

[97] Wu G. (2013). Plant microRNAs and development. J. Genet. Genom..

[98] Li C., Zhang B. (2016). MicroRNAs in control of plant development. J. Cell. Physiol..

[99] Wang J.-W., Czech B., Weigel D. (2009). miR156-regulated SPL transcription factors define an endogenous flowering pathway in Arabidopsis thaliana. Cell.

[100] Jung J.-H., Lee S., Yun J., Lee M., Park C.-M. (2014). The miR172 target TOE3 represses AGAMOUS expression during Arabidopsis floral patterning. Plant Sci..

[101] Nair S.K., Wang N., Turuspekov Y., Pourkheirandish M., Sinsuwongwat S., Chen G., Sameri M., Tagiri A., Honda I., Watanabe Y. (2010). Cleistogamous flowering in barley arises from the suppression of microRNA-guided HvAP2 mRNA cleavage. Proc. Natl. Acad. Sci. USA.

[102] Cao D., Wang J., Ju Z., Liu Q., Li S., Tian H., Fu D., Zhu H., Luo Y., Zhu B. (2016). Regulations on growth and development in tomato cotyledon, flower and fruit via destruction of miR396 with short tandem target mimic. Plant Sci..

[103] Wang T., Ping X., Cao Y., Jian H., Gao Y., Wang J., Tan Y., Xu X., Lu K., Li J. (2019). Genome-wide exploration and characterization of miR172/euAP2 genes in Brassica napus L. for likely role in flower organ development. BMC Plant Biol..

[104] Zheng G., Wei W., Li Y., Kan L., Wang F., Zhang X., Li F., Liu Z., Kang C. (2019). Conserved and novel roles of miR164-CUC 2 regulatory module in specifying leaf and floral organ morphology in strawberry. New Phytol..

[105] Ma Z., Jiang J., Hu Z., Lyu T., Yang Y., Jiang J., Cao J. (2017). Over-expression of miR158 causes pollen abortion in Brassica campestris ssp. chinensis. Plant Mol. Biol..

