# Supplementary material for: Circular RNAs Repertoire and Expression Profile during Brassica rapa Pollen Development
Source: Int J Mol Sci. 2021 Sep 24;22(19):10297. doi: 10.3390/ijms221910297 (PMC8508787; doi:10.3390/ijms221910297)

Supplementary document

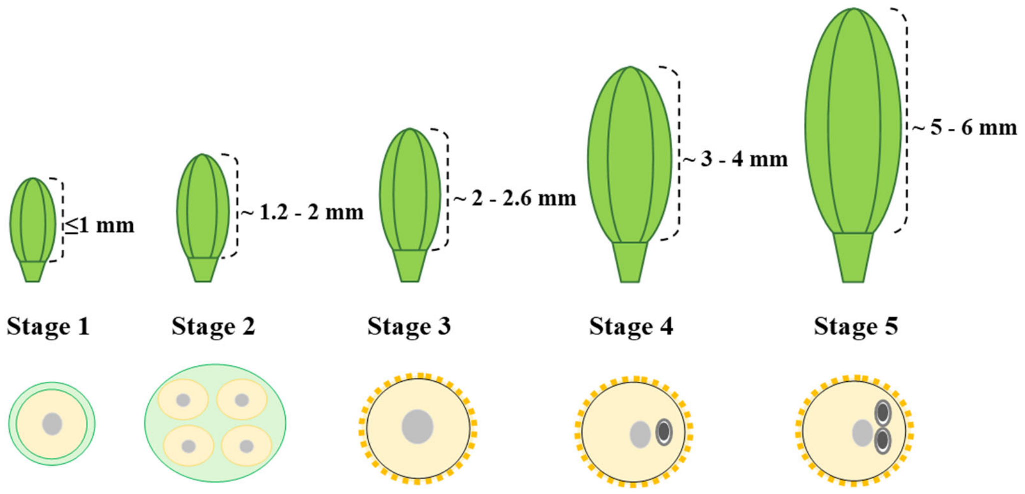

Figure S1. A schematic representation of floral buds at different developmental stages and their corresponding microspores. Stage 1: pollen mother cells, stage 2: tetrad, stage 3: uninucleate pollen, stage 4: binucleate pollen, and stage 5: mature pollen.

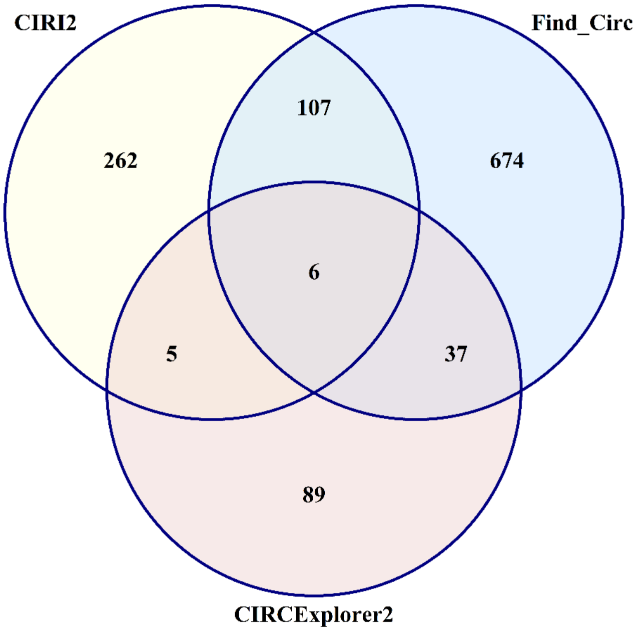

Figure S2. A Venn diagram of unique and shared circRNAs predicted by three algorithms: CIRCexplorer2, find_circ, and CIRI2.

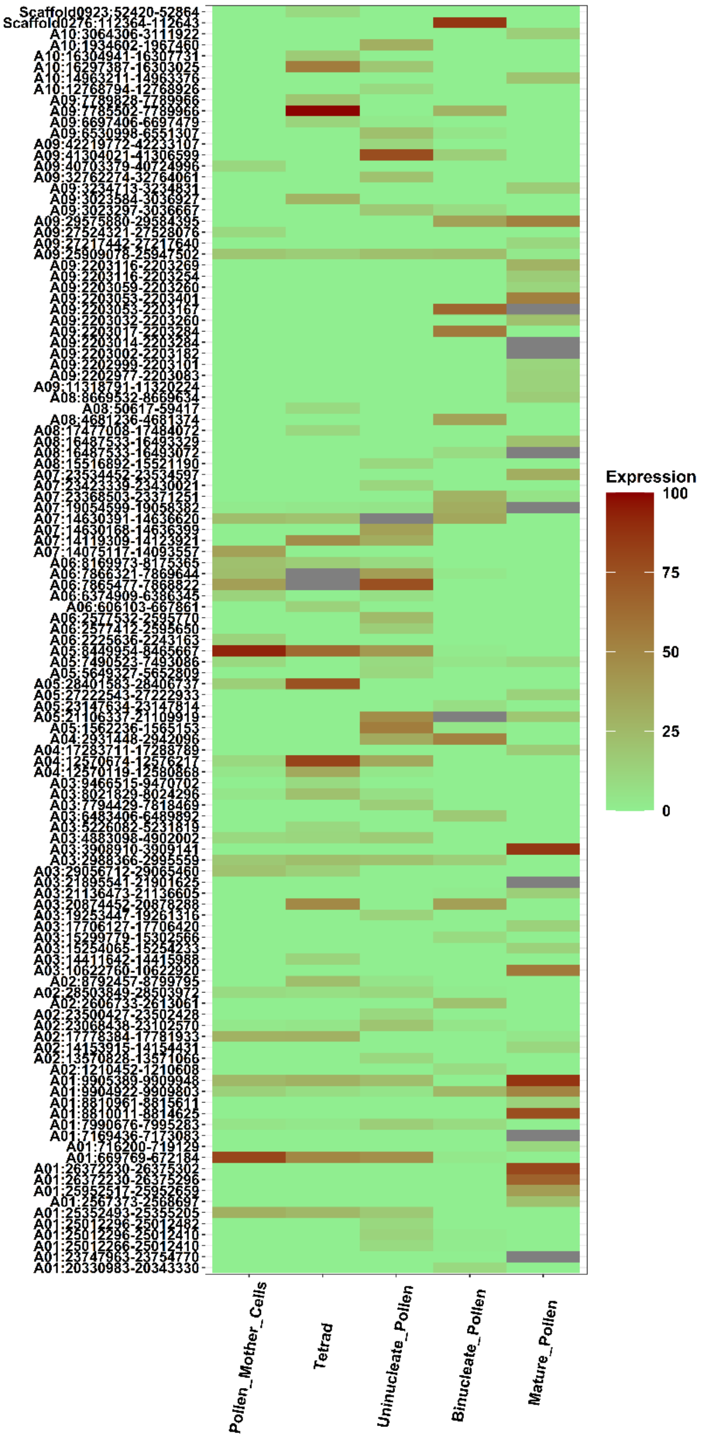

Figure S3. A heatmap showing the expression pattern of the top 60 ($\alpha \geq 0.001$) differential expressed circRNAs during pollen development in *B. rapa*. Gray tiles represent the expression of more than 100.

The nucleotide sequence of validated circular RNAs' junction

[F:] Forward primer, [R:] Reverse primer

circRNA ID: A05:24735621-24735783

Gene name: BraA05g034940.3C

Exon 3: A05:24735622-24735783

TGTTTCCTTCCATCCAACCAGGGAAAGGGTCTTTGTAAGTGCTTTCGATGAC[GCTAGGCCTTATAATAACCAC:R]
AGGTACGTCC[CCTCTAGTGCTATTGATCATC:F]ATTTCTCCCATTGCTTTTGTGAAGACATAAGTGTCTTGCCATC
CATATGATCTCGCCC

Exon 4: A05:24735849-24736050

TCTCTAGACCGAGATCCTTCATCTTCTGCGCCTCCTCTTGATCTTGAGTACCTTCTCTTGCAGCATCAAGAGCTAG
CTTCAACTCTTTAGCAACATCTAATGCTTTCCTGTTTCCTTCAAGGAAGTTCTCGGTTGCTATACAATCTCCCATT
GAGAATGGCTTCTCCATGATCCTTCCTTGTCTTTGTCCATTCACATAAG

Circular RNA junction:

[CCTCTAGTGCTATTGATCATC:F]ATTTCTCCCATTGCTTTTGTGAAAACATAAGTGTCTTGCCATCCATATGATCT
CGCCCTCTCTAGACCCTTTGTACGTGCTTTCGATGAC[GCTAGGCCTTATAATAACCAC:R]

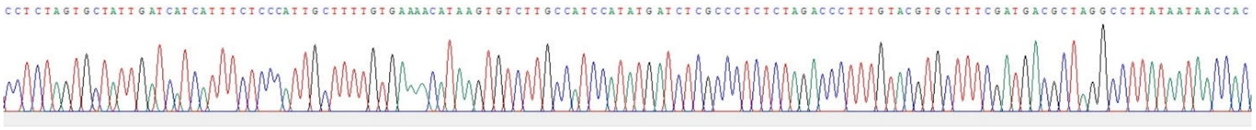

---

circRNA ID: A03:5712452-5713124

Gene name: BraA03g013120.3C

exon2: A03:5712453-5712557

CTTGGATATGGTAAGTGCAGGGCTCCACTGCTCTTTCAGAATGTCAAGACAGATACTGCCGTTACTGTTGACATT
CGGGTG[GTAAACCTTGGTGCGGAAAGA:R]AAC

exon4: A03:5713030-5713099

AGC[ACTGCATGAGACAGGAGGATC:F]CCTTTGGAGGTCCCTAAGCTCCTTGTTGATTCTTTTCGAAGCCAT

Intron from end of exon 4:

CTTGAACTGTACTCGCTGCTTCTAG

Circular RNA junction:

[ACTGCATGAGACAGGAGGATC:F]CCTTTGGAGGTCCCTAAGCTCCTTGTTGATTCTTTTCGAAGCCATCTTGAAC
TGTACTCGCTGCTTCTAGCTTGGATATGGTAAGTGCAGGGCTCCACTGCTCTTTCAGAATGTCAAGACAGATACT
GCCGTTACTGTTGACATTCGGGTG[GTAAACCTTGGTGCGGAAAGA:R]

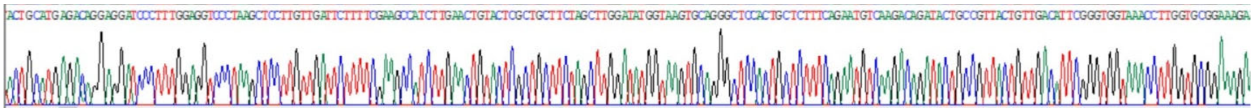

circRNA ID: A01:29811-30193

Gene name: BraA01g000090.3C

Exon 3: A01:29812-29873

TTCAACAACCTCCAGCTGACTCGGACTCTCTTACCAAT[GAAACAACTTCTTCTCCTGCC:R]AG

Exon 5: A01:30139-30193

CC[CCTGCATGAGATCACCTTTTC:F]CACACTAGACAAACCTAAACTCCTTTTGCAG

Exon 6: A01:30486-30593

TTAACTGCATTGCTTGCTGAGCTTGGGCTGAATATTCAAGAGGCGCATGTTTTTCTACAACCGATGGCTTCTCAC
TCGATGTTTTTGTCGTTGATGGTTGGTACGAGG

Circular RNA junction:

[CCTGCATGAGATCACCTTTTC:F]CACACTAGACAAACCTAAACTCCTTTTGCAGTTAACTGCATTGCTTGCTGAG
CTTGGGCTGAATATTCAAGAGGCGCATGTTTTTCTACAACCGATGGCTTCTCACTCGATGTTTTTGTCGTTGATG
GTTGGTACGAGGTTCAACAACCTCCAGCTGACTCGGACTCTCTTACCAAT[GAAACAACTTCTTCTCCTGCC:R]

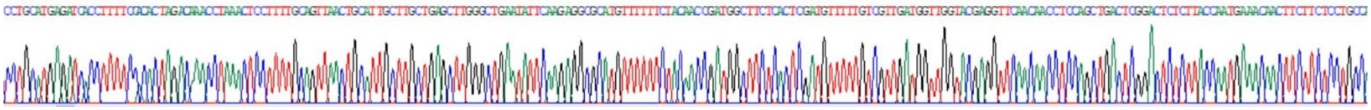

---

circRNA ID: A06:3536171-3536581

Gene name: BraA06g006120.3C

Exon 7: A06:3536172-3536258

TTGGGCTTCCATTCACCCATGTACTCAGGGTTGGGAATGGTAGGG[GCAGTCCACTCACCATCTTCT:R]TCATCAT
CCCAGTCCTCAGG

Exon 9: A06:3536555-3536813

[TTCTTGGCGTTGGGGTCCTTG:F]ATCTTCTTGGGTGGGAGAAGATCCCAGTCAGAGTACAGGCTACCGGTTTGCT
TCTCAACATTGTCAATGAGAATGGTGTAAGTAGCATCTGGGCGGAGGATGAACGTGTAGACATGGGTGAGCTGG
TCAGTTTCACACGGAACATCCTTCTTGATCAGGTGGTTGGCGTCATTGTAGGTAAGGATAGCATGCACTTTCTTC
GTGGTGTACCCACAGATATCGGGACCAAACATGATG

Circular RNA junction:

[TTCTTGGCGTTGGGGTCCTTG:F]ATTTGCTTGGGCTTCCATTTGGGCTTCCATTCACCCATGTACTCAGGGTTGGG
AATGGTAGGG[GCAGTCCACTCACCATCTTCT:R]

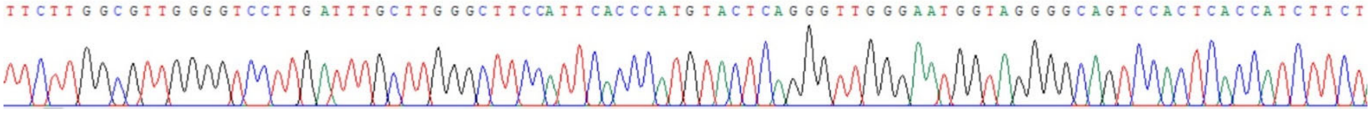

circRNA ID: A08:20496640-20496925

Gene name: BraA08g030040.3C

Exon 14: A08:20496470-20496710

AGCTGAAGCGGTTCCCAACTCTAGCAAGTGATATAGCAGCTGCTGCAAACGAAGCTCTCGAAAGATTCAGAGAC GAAAGCAGGAAAACGGTTCTGCGTCTCGTGGACATGGAATCCAGCTACCTCACGGTTGAGTTCTTCAGAAAACT TCACCTTGAGCCAGAGAAAGAGAAACCAAACCAGC[GAAATGCTCCACCACCAAAC:R]GCAGACATCCACTCCG ATAATCACTTCAGAAAGATCG

Exon 15: A08:20496786-20496925

ATCCAACGTGAGTGCGTACATAAACATGGTCTGTGACACATTGAGAATTTCTCTTC[CCAAAGCTGTTGTTTACTG C:F]CAAGTTAGAGAAGCTAAGAGATCGCTCCTCAACTTCTTCTACGCTCAAGTTGGCAGGAAAGAG

Circular RNA junction:

[CCAAAGCTGTTGTTTACTGC:F]CAAGTTAGAGAAGCTAAGAGATCGCTCCTCAACTTCTTCTACGCACTTCAGAA AACTTCACCTTGAGCCCGAGAAAGAGAAACCAAACCAGA[GAAATGCTCCACCACCAAAC:R]

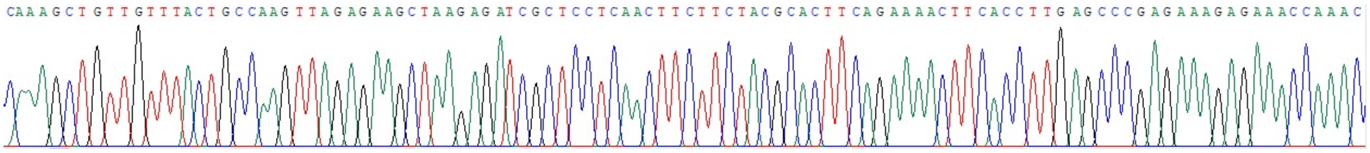

---

circRNA ID: A03:16540782-16540980

Gene name: BraA03g033430.3C

Exon 1 A03:16540030-16540800

TATTGGGGTAAGTTGGTCACTGGACCACTGCAAGCTTTAGGGCTCTGAGTTCCCATGAGTTTGGGGCTCACGTTT GAGCACTGGAAAGTCGCTGGACCATCTGCTCCGCTGTACCTAATGTCAATGTCTCCAATCTCAACGTTTTGGCAT GGGTATCC[CCTGCTGCACAATAGCTTCAC:R]TGCATCCTTGTTCCCTGATGTTCCTCTGATCTGCTTGAAGCTGAT GTTGGCAAGCTTAATCGTTGATGGTTTCTGCTTGTTACATTGGTTCCAAGGGCAGTACT………..

Exon 2: A03:16540898-16541203

ATGGGAAGTTTCTTGCAGTTGAAAGTCTGGTGACAGTTGTTAACTCTCCAAGCTGCGTTTCCTTCACCATCGAAA GCTCCACCTCCGTTCAACTTAAAGCCATTAATGTTGCCAAAGACAATCCATGTGTCCTTTCCCTGGATCGCGTTAC CATCAGCTTTGACAGTGCCTTGAATGGTGATCTCGACTGGAGCTTTGCATGGACCC[CTCATCTGGATCTCACCAA GC:F]TTGAACTCTCCTTTTGGGATCACCACTCTGCTCGGTGTTGGAGATTGGCATGCTGTTGTAAATGCTTTCAGA AGTGC

Circular RNA junction:

[CTCATCTGGATCTCACCAAGC:F]TTTAGGGCTCTGAGTTCCCATGAGTTTGGGGCTCACGTTTGAGCACTGGAAA GTCGCTGGACCATCTGCTCCGCTGTACCTAATGTCAATGTCTCCAATCTCAACGTTTTGGCATGGGTATCC[CCTG CTGCACAATAGCTTCAC:R]

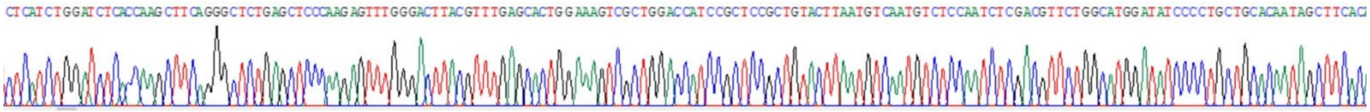

circRNA ID: A03:15254021-15254249

Gene name: BraA03g030350.3C

Exon 1: A03:15253909-15254319

TAGAGAGAGAAGAAGAAGAATCCGACGGCGGCAACAGTGCCGGCGATGGAGAGCTTCACATTAGTGGCGCTGC CGTCGAGTACCTCGGGGCCGGGGGTGGGTGCATCGGAAGATGGTCCATCAACAACCTCTGGGGTAGGTGCTGGT GGGGAGGAGACGGTGGGTCCCTCGGCAGATTCACTCGG[GCTGGTTGCTGCGTAATCGTC:R]TTCGGCTGTTGGT TCCTCTGCCACAGGGG[ACGATGCTTTGGGTGCTGAGG:F]CTGACGATGATTTGGGTGCAGCAGCCGGTG CCTTGGTCTGCGTGGTGGGTGCTTTTGTAGGGGAGGCGGTGGGAGCAGCTGAGGGTGCCTCGGCAGCG AATGCGGTGGCCACGACCAAGGCCAATAGAGCCAATACGACAAATTGACGTGCCAT

Circular RNA junction 1:

[ACGATGCTTTGGGTGCTGAGG:F]CTGACGATGATTTGGGTGCAGCAGCCGGTGCCTTGGTCTGCGTGG TGGGTGCTTTTGTAGGGGAGGCGGTGGGAGCAGCTGAGGGTGCCTCGGAAGATGGTCCATCAACAACCT CTGGGGTAGGTGCTGGTGGGGAGGAGACGGTGGGTCCCCCGGCAGATTCACTCGG[GCTGGTTGCTGCGTAATCG TC:R]

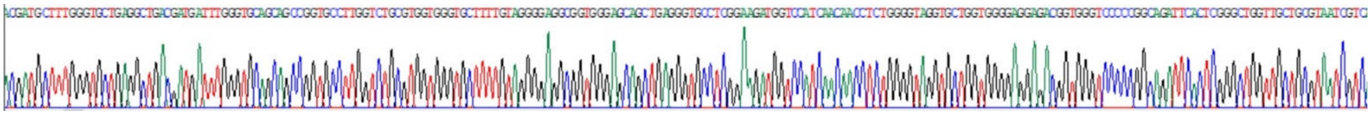

---

circRNA ID: A03:1972950-1973522

Gene name: BraA03g004570.3C

Exon 4: A03:1972662-1972846

TCTCTTCCTGAGACATGCCAGAGTGAATGCATATCGAGGGGAAATTGCATTCGATAAGCAACTTGTTCAGCTCCC CAGCTCTGCTAACGCTCTTCACAAAGATAACAACTTGATTGAAGTCCAATGCATCGAGAAGGTCATTCAACTTGC GGTTTTTCTCCATCTCGCTCAGTTTGATGTAGTG

Exon 5: A03:1972951-1973007

TGGACAAGTCCATGAAGAGTCAATTTCGCTTCAT[CATCAACATAAATCTCCATTG:R]G

Exon 6: A03:1973483-1973601

[TCTTGCATAAATTTCTTGCAG:F]ACAGGGCGTATCTCTTTGCTGAGCGTTGCTGAGAACATCATAACTTGTTTGTC GTGAGGAGTCATCTTGAAAATCTCCTGCACATCCCTCCGCATGT

Circular RNA junction:

[TCTTGCATAAATTTCTTGCAG:F]ACAGGGCGTATCTCTTTGCTGAGCGTTGCTGAGAACATCATAACTTGTTTGTC GTGAGGAGTCATCTTGATTTTCTCCATCTCGCTCAGTTTGATGTAGTGCTGGACAAGTCCATGAAGAGTCAATTT CGCTTCAT[CATCAACATAAATCTCCATTG:R]

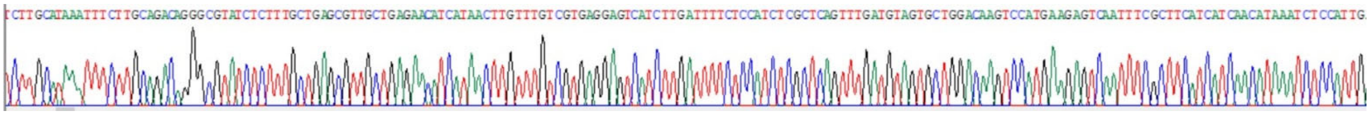

circRNA ID: A06:1190076-1190251

Gene name: BraA06g001990.3C

Exon 4: A06:1190077-1190136

CTTGTTCTTCAGTGTACTTAGATTCATCATCTTTGATC[TGTTTAAGACCTCCAATGGCAG:R]

Exon 5: A06:1190204-1190251

TTCAAAT[TCACGTTCCATCTCTTCAAGC:F]ATAGTCTTCCTCTTTGCAGCTGT

Circular RNA junction:

[TCACGTTCCATCTCTTCAAGC:F]ATAGTCTTCCTCTTTGCAGCTGTATCATCTTTGATC[TGTTTAAGACCTCCAATGGCAG:R]

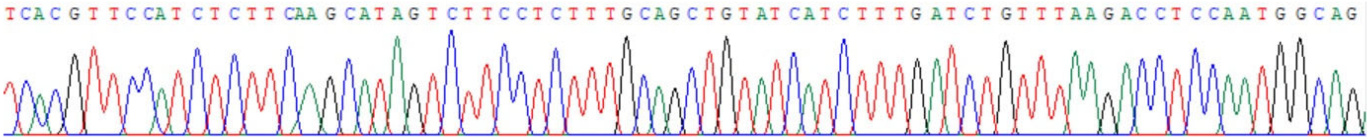

Supplement: Supplementary file 1 [file ijms-22-10297-s001.zip › Supplementary Document.pdf]
